# P-1142. Evaluating Ambulatory Antibiotic Prescribing for Respiratory Infections at a Pediatric Institution

**DOI:** 10.1093/ofid/ofae631.1329

**Published:** 2025-01-29

**Authors:** Diana Yu, Louise E Vaz, Dawn Nolt

**Affiliations:** Doernbecher Children's Hospital/OHSU Healthcare, Portland, Oregon; Doernbecher Children's Hospital/OHSU, Portland, Oregon; Oregon Health and Science University/Doernbecher Children’s Hospital, Portland, OR

## Abstract

**Background:**

Over 85% of antibiotic prescribing occurs in outpatient settings such as emergency departments (ED), urgent care centers (UCC), and primary care clinics (PCC); almost half of antibiotics are prescribed for respiratory infections (RI). Given the rise of telehealth visits (TH) in both UCC and PCC settings in the past several years, this study examines the effect of location sites on antibiotic prescribing for RI at our institution.

**Figure:**
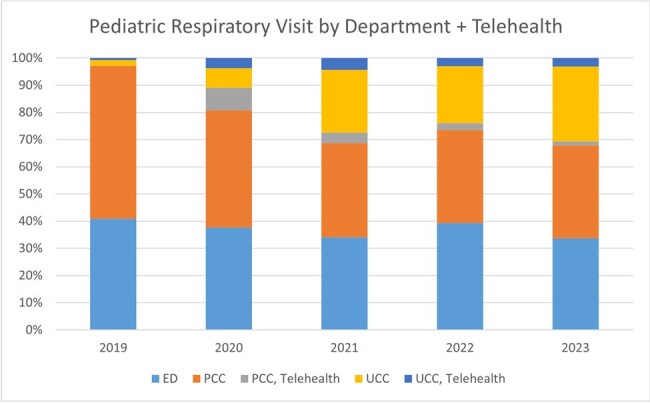

**Methods:**

Single-center, retrospective cohort study in Portland, Oregon, USA. Patients < = 20 years of age seen in the ED, UCC, and PC settings between 1/1/2019-12/31/2024, were included. Electronic data included age, race, ethnicity, location and encounter type, payor status, visit ICD-10 codes, and antibiotic prescription details. Multivariate logistical regression determined the effect of patient factors on antimicrobial prescribing.

**Figure:**
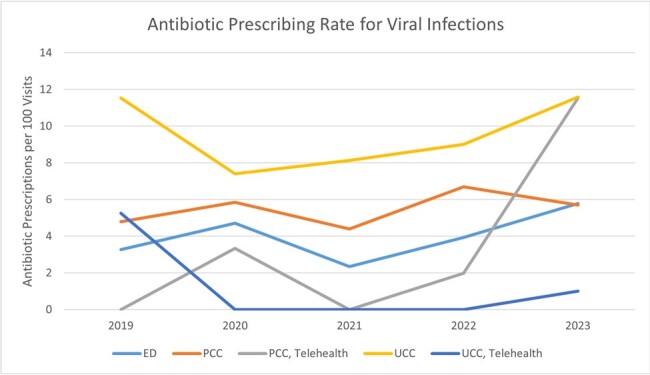

**Results:**

A total of 157,499 ambulatory encounters were included of which 24,236 were respiratory ICD10 code-related visits. Respiratory visit rates were not significantly different between non-Hispanic White and Hispanic/non-White children or by age group. RI were categorized as viral (57.7%), possible bacterial (37.6%), or co-infection (4.7%); antibiotics were prescribed 5.3%, 60.4%, and 62.8% of visits respectively. The rate of antibiotic prescribing for viral and possible bacterial RI was not different between non-Hispanic White and Hispanic/non-White children or between payor status. For viral RI, PCC and UCC settings were more likely to prescribe antibiotics compared to the ED (OR 1.4 and OR 2.36, respectively). Furthermore, PCC and UCC were less likely to prescribe narrow-spectrum antibiotics for possible bacterial RI compared to the ED (OR 0.84 and OR 0.79, respectively), including TH PCC and TH UCC visits (OR 0.29 and OR 0.56, respectively).

**Figure:**
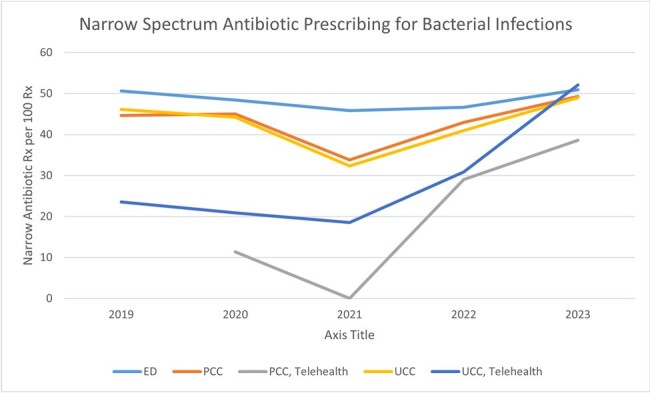

**Conclusion:**

Pediatric patients with RI can receive ambulatory care through multiple settings. PCC TH (although a small percentage of all RI visits) shows the highest use of antibiotics for viral RI and the least narrow spectrum antibiotic prescribing for presumed bacterial RI. Efforts to reduce antibiotic use in viral RI should focus on non-ED settings, including TH. Strategies to encourage use of narrow-spectrum activity agents for RI should be shared with all departments.

**Disclosures:**

**All Authors**: No reported disclosures

